# Primary Cutaneous Vasculitis Masquerading as Drug Induced following Aspirin Desensitization

**DOI:** 10.1155/2013/745714

**Published:** 2013-11-24

**Authors:** Maryam Ali Al-Nesf, Hassan M. S. Mobayed, Tasleem Raza

**Affiliations:** Division of Respiratory, Department of Medicine, Hamad General Hospital, Hamad Medical Corporation, P.O. Box 3050, Doha, Qatar

## Abstract

Aspirin-exacerbated respiratory disease (AERD) is a well-known clinical condition. Aspirin desensitization followed by daily aspirin therapy is the treatment of choice. We report a challenging case of primary cutaneous vasculitis following aspirin desensitization in a patient with AERD. The vasculitis was likely suppressed with higher dose systemic steroid use to control asthma. Aspirin desensitization led to improved asthma control and steroid reduction, which led to manifestation of prior suppressed cutaneous vasculitis. In our case, there was no evidence of systemic involvement and the patient had a favorable outcome with appropriate therapy.

## 1. Introduction

Aspirin desensitization is one therapeutic modality for patients with aspirin-exacerbated respiratory Disease (AERD). It may precipitate multiple side effects including cutaneous vasculitis (CV) [1, 2]. CV may be primary in nature or secondary to other multiple underlying disorders such as systemic necrotizing vasculitis, connective tissue diseases, infections, or malignancies [3]. Drug induced cutaneous vasculitis (DIV) has been reported secondary to nonsteroidal anti-inflammatory drugs (NSAIDs) [4]. 

Here we report a challenging case of primary cutaneous vasculitis following aspirin (ASA) desensitization in a patient with AERD. Previous reports found a relation between autoimmune vasculitis and aspirin-induced asthma (AIA) [5, 6]. In our case, there was no systemic involvement. The patient was treated with colchicine and had a favorable outcome.

## 2. Case Report

A 25-year-old gentleman was referred with a difficulty in controling asthma. He had history of chronic rhinosinusitis, recurrent nasal eosinophilic polyps, and two previous polypectomies. Asthma was exacerbated by Ibuprofen in the past. He required two asthma exacerbation related hospital admissions in one year. Examination showed cushingoid feature and steroid related skin changes. Lung exam was significant for diffuse wheezing. No nasal polyps were evident on exam. Other systemic examinations were normal. Erythrocyte sedimentation rate (ESR) was normal. Serum total IgE was 284 KU/L (normal 0–114). Blood eosinophils were 600. Skin prick test was positive for dog and horse hair and spirometry showed moderate reversible obstruction. Chest radiographs showed no abnormalities.

His asthma was categorized as moderate to severe with score of 10 on asthma control test (ACT). He was active smoker but managed to quit; however, this did not improve his asthma symptoms. We offered aspirin desensitization.

A two-day aspirin (ASA) desensitization protocol was carried out using Stevenson and Simon regimen [7]. ASA challenge was performed in the first day as part of the desensitization regimen and the patient developed difficulty of breathing, wheezing, and drop in FEV1 by 30% confirming the presence of sensitization. Patient was successfully maintained on 600 mg ASA twice daily. Asthma symptoms improved remarkably and ACT score was 17 three weeks later. Prednisolone was successfully tapered to 10 mg. On day 26, patient developed generalized maculopapular erythematous itchy skin rash, progressing to nonblanching purpuric lesions. All work-up for systemic vasculitis was negative including immunological tests and a clinical diagnosis of aspirin induced cutaneous vasculitis was made without skin biopsy. 

Aspirin was discontinued and the steroid dose was increased, leading to significant improvement in vasculitic rash. There were no new lesions over next 10 days. Montelukast was questioned as a cause of CV [8] and discontinued. Then, corticosteroid dose was reduced while patient was off ASA to uncover any steroid suppressed primary vasculitis. At 10 mg prednisolone, the patient again developed vasculitic rash. Skin biopsy confirmed the diagnosis of primary cutaneous vasculitis. It showed the presence of neutrophilic leukocytoclastic vasculitis. No granuloma, necrotizing vasculitis, or tissue eosinophilia could be found. No evidence of Churg-Strauss syndrome clinically or histologically. Patient was started on colchicine that resulted in CV resolution.

## 3. Discussion

Aspirin-exacerbated respiratory disease (AERD) is a condition characterized by the presence of nasal polyps, chronic hypertrophic eosinophilic sinusitis, asthma, and sensitivity to cyclooxygenase-1 (COX-1) inhibiting drugs, namely, ASA and other NSAIDs. Few terms have been used to describe Aspirin-exacerbated respiratory disease (AERD). These include Samter's triad, Aspirin Induced Asthma (AIA), aspirin sensitive asthma and aspirin hypersensitivity. AERD was first described in 1922 by Widal et al; however, the pathophysiology is not fully understood. The reaction is not IgE-mediated but patients can present with anaphylaxis after exposure to COX-1 inhibiting drugs. Aspirin challenge is the gold standard for diagnosing AERD [1]. 

Aspirin desensitization, followed by continuous treatment with ASA, is an effective treatment for patients with AERD in whom conventional therapy has failed [9]. The exact mechanism leading to efficacy of ASA desensitization is not yet well clarified. Few biomarkers have been shown to correlate with success, including exhaled nitric oxide level and sputum tryptase level during the desensitization process. Sputum IL-4 and matrix metalloproteinase-9 (MMP-9) reductions have been noted after 6 months of aspirin treatment [10]. Most patients tolerate ASA well and only few require discontinuation due to side effects. In a study of 172 patients with AERD who had ASA desensitization, 24 out of 172 patients (13%) discontinued ASA during the first year because of side effects. In this study, only 6 patients had ASA induced urticaria, but no one developed CV [2].

Cutaneous vasculitis is a clinical condition with specific histological features. Many terms have been used to describe this condition. Drug induced vasculitis should always be considered in any patient presenting with CV. Drugs are associated with approximately 10–15% of all vasculitic dermatologic lesions. DIV is a diagnosis of exclusion. The interval between the first exposure and appearance of symptoms varies from hours to years [11]. NSAIDs including COX-2 selective inhibitors have been reported to precipitate CV in multiple case reports [3, 4]. However, there is no report of cutaneous vasculitis precipitated by ASA. 

Autoimmune CV was also reported in AIA [5, 6]. The exact mechanism to explain the pathogenicity of vasculitis remains unknown. Many immunological findings have been proposed to explain the pathophysiology of vasculitis associated with AIA. Distinct deposits of IgG, IgA, IgM, C3C, and fibrinogen may be seen in the biopsy of patients with vasculitis and AIA, which indicate that an active immunologic process is present [6]. 55% of AIA patients have serum antinuclear antibodies at a low titer and 6% of all AIA have evident clinical signs of autoimmune disease [7]. However, in our patient the autoimmune markers were all negative and vasculitis was limited only to skin.

Montelukast was a potential etiology of vasculitis although a true relationship between systemic vasculitis and the use of Montelukast was not clearly justified [8]; however, our patient developed vasculitis despite discontinuation of Montelukast and so this was unlikely to be the culprit.

ASA induced vasculitis was a strong consideration because of the temporal relationship between occurrence of vasculitis and aspirin use in our patient with AERD. The patient had recurrence of symptoms despite discontinuation of potential offending medication, leading to the final diagnosis of primary cutaneous vasculitis.

In summary, our case was challenging as patient did not have any prior evidence of vasculitis, developed symptoms following ASA desensitization while being treated with ASA. Physician should remain vigilant to the emergence of new symptoms and findings during change in asthma therapy, in particular the reduction of systemic steroids with improved asthma control, which may unmask underlying immunological disorders.

## Abbreviations

AERD: Aspirin-exacerbated respiratory disease
CV: Cutaneous vasculitis
DIV: Drug induced cutaneous vasculitis
NSAIDs: Nonsteroidal anti-inflammatory drugs
ASA: Aspirin
ESR: Erythrocyte sedimentation rate
ACT: Asthma control test
COX-1: Cyclooxygenase-1
COX-2: Cyclooxygenase-2
MMP-9: Matrix metalloproteinase-9.
